# Basic Study for Ultrasound-Based Navigation for Pedicle Screw Insertion Using Transmission and Backscattered Methods

**DOI:** 10.1371/journal.pone.0122392

**Published:** 2015-04-10

**Authors:** Ziqiang Chen, Bing Wu, Xiao Zhai, Yushu Bai, Xiaodong Zhu, Beier Luo, Xiao Chen, Chao Li, Mingyuan Yang, Kailiang Xu, Chengcheng Liu, Chuanfeng Wang, Yingchuan Zhao, Xianzhao Wei, Kai Chen, Wu Yang, Dean Ta, Ming Li

**Affiliations:** 1 Department of Orthopedics, Changhai hospital affiliated to the Second Military Medical University, Shanghai, PR China; 2 Department of Orthopedics, 401st Hospital Center, Qingdao, Shandong Province, PR China; 3 Graduate Management Unit, Changhai hospital affiliated to the Second Military Medical University, Shanghai, PR China; 4 Department of Electronic Engineering, Fudan University, Shanghai, PR China; Toronto Western Hospital, CANADA

## Abstract

The purpose of this study was to understand the acoustic properties of human vertebral cancellous bone and to study the feasibility of ultrasound-based navigation for posterior pedicle screw fixation in spinal fusion surgery. Fourteen human vertebral specimens were disarticulated from seven un-embalmed cadavers (four males, three females, 73.14 ± 9.87 years, two specimens from each cadaver). Seven specimens were used to measure the transmission, including tests of attenuation and phase velocity, while the other seven specimens were used for backscattered measurements to inspect the depth of penetration and A-Mode signals. Five pairs of unfocused broadband ultrasonic transducers were used for the detection, with center frequencies of 0.5 MHz, 1 MHz, 1.5 MHz, 2.25 MHz, and 3.5 MHz. As a result, good and stable results were documented. With increased frequency, the attenuation increased (*P*<0.05), stability of the speed of sound improved (*P*<0.05), and penetration distance decreased (*P*>0.05). At about 0.6 cm away from the cortical bone, warning signals were easily observed from the backscattered measurements. In conclusion, the ultrasonic system proved to be an effective, moveable, and real-time imaging navigation system. However, how ultrasonic navigation will benefit pedicle screw insertion in spinal surgery needs to be determined. Therefore, ultrasound-guided pedicle screw implantation is theoretically effective and promising.

## Introduction

Scoliosis is a complicated three-dimensional deformity, and idiopathic scoliosis generally develops in adolescents with an incidence of 0.75–2.4%[1, 2]. In patients with severe scoliosis, surgery is one of the few effective treatments[3]. Posterior pedicle screw fixation is a widely used procedure that immobilizes segments of the vertebral column with large screws. However, the fixation is associated with a risk of injury to the regional neural and vascular structures[4]. It has been reported that the misplacement rate of thoracic pedicle screws is 6.0–54.7%[5].

To improve the accuracy of screw placement, auxiliary surgical devices have been developed such as the C-arm X-ray machine, computer-assisted surgery(CAS),and neurophysiological monitoring. The benefit of radiography is that it produces real time images of the position of the pedicle screws but exposure to radiation can be detrimental to the surgeon’s health[6]. Moreover, two-dimensional images do not provide adequate spatial accuracy[7]. CAS provides clear three-dimensional images and has significantly reduced the rate of screw misplacements[8], but it is expensive to use, occupies a large space, and requires complicated registration and tracking methods[9, 10]. Finally, neurophysiological monitoring demands strict sterile conditions in the operating room and the tacit cooperation of the surgical staff[11].

Therefore, it is necessary to develop an imaging technique for pedicle screw fixation surgery that uses free ionizing radiation, is affordable, and has a low complication rate. Quantitative ultrasound(QUS) possesses these features, however, studies needs to be conducted to determine the safety and efficacy of QUS[12].

Ultrasonic signals attenuate in bone and reflect with a large acoustic energy at the interface of the soft and bone tissue[13]. As such, the bone creates a blind spot for ultrasound at high frequencies. Furthermore, using lower frequencies might lead to considerable loss in the resolution of the images[14]. Therefore, a sufficient understanding of the acoustic properties of human vertebral cancellous bone and adequate understanding of the appropriate frequencies might help promote ultrasonic penetration while preserving image resolution.

During the preparation of the screw hole, A-mode images might provide guidance for the surgeons by showing warning waves of the oncoming perforation[15], and backscattering technology is a good method that adopts the physical properties of acoustic impedance. When the wavelength of the incident wave is smaller than the diameter of the interface, it creates reflectance, but when the incident wave is longer than the diameter of the interface, it results in scattering. The waves, which are reflected 180°to the incident wave, are called backscattering waves and are highly energetic[16, 17]. When backscattering signals are received, the amplitudes of the A-mode images are abnormally amplified and it might be possible to use this to predict the distance between the interface of the cancellous and cortical bone[18].

Therefore, the focus of this study was to better understand the acoustic properties of human vertebral cancellous bone using transmission measurements and to ascertain the warning distance by analyzing the backscattering measurements.

## Materials and Methods

### Spine specimens

With ethical approval granted by the Committee on Ethics of Biomedicine Research at the Second Military Medical University,14fresh human lumbarvertebral specimens were tested. The specimens were disarticulated from 7un-embalmed cadavers(4 males, 3 females, 73.14 ± 9.87 years, range: 59–85 years, two specimens from each cadavers)provided by the anatomy department at the Second Military Medical University. Written informed consent was obtained from the donor or the next of kin for the use of this sample in the current research. To account for variability in porosity and bone mineral density, the samples were taken from randomized spinal levels; 7specimens from7different cadavers were used for the transmission measurements, and the other 7were used for the backscattering measurements.

For specimen preparation, the surrounding soft tissue and the vertebral arch of the specimens were removed using a scalpel, followed by the removal of the anteroposterior cortical plates of the vertebral body using a hacksaw. For the transmission measurements, the anteroposterior thickness of the cancellous bone was about 0.9cm. For the backscattering measurements, 3–6 mm of the ventricular bodies was sectioned in the dorsal to abdominal direction and the sections were measured with calipers (Table 1).

**Table 1:** Thickness of the specimens after resection.

Specimen	Thickness, cm					
	Ⅰ	Ⅱ	Ⅲ	Ⅳ	Ⅴ	Ⅵ
1	2.40	1.60	1.20	0.90	0.70	0.40
2	1.80	1.50	1.00	0.40		
3	1.90	1.40	0.90	0.70	0.40	0.20
4	2.00	1.50	1.10	0.90	0.60	0.30
5	2.00	1.20	0.90	0.40		
6	2.60	1.90	1.50	0.90	0.60	0.30
7	2.60	2.00	1.20	0.90	0.60	0.40

The exposed axis of the specimens was matched with the cranio-caudal anatomical axis, as this has been shown to represent the highest acoustic properties in the vertebral cancellous bone[19]. After being defatted with a water jet, the cancellous bone was immersed in amethanol/isopropyl solution(that was frequently changed)for several days and then stored in deionized water that had a small quantity of methanol added to reduce the surface tension of the water and to eliminate air bubbles[20]. We confirmed that all air bubbles were removed by comparing the results of the QUS measurements with vacuum degassing.

### Ultrasonic Measurements

**Fig 1:**
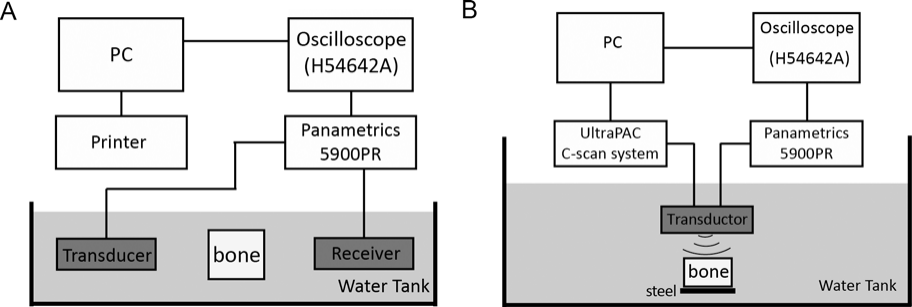
Experimental Systems. An acoustic water bath was used to measure the speed of sound, broadband ultrasound attenuation, and backscatter coefficients. The ultrasonic system for transmission and backscattering measurements is shown in (A) and (B) respectively.

An ultrasonic immersion system was applied in the current investigation and is shown in Fig 1A and 1B. The water tank was filled with deionized water maintained by a canister filter(Magnum 350, Marineland, Moorpark, California) and an integrated filtration system and all experiments were performed in immersion at room temperature. By observing an absence of scatter using pulse-echo measurements of the water medium, it was assured that the filtration system did not pass millimeter-sized air bubbles into the water bath.

The position of the bone specimen was computer-controlled by an UltraPAC C-scan system (Physical Acoustics Corp., NJ, USA) using four stepper motors that controlled the X, Y, and Z translations and rotations. A custom specimen holder clamped the bone samples between three thin, equi-spaced needles embedded in a Plexiglas ring. This system was used to provide adequate stability during sample translation and rotation while minimizing the absolute contact area of the specimen within the holder(thereby eliminating undesired artifacts). Each specimen was raster scanned in the XZ plane through a step size of 2 mm in both directions.

The acoustic properties were measured using unfocused broadband ultrasound transducers: a pair of0.5 MHz (0.5 inch diameter), 1 MHz (0.5 inch diameter), 1.5 MHz (0.375 inch diameter), 2.25 MHz (0.25 inch diameter), and 3.5 MHz (0.25 inch diameter) center frequency transducers (Olympus Corp., MA, USA). Pulse signals were applied using a scan system and adjusting the transducer to get the maximum amplitude of the signal. The broadband ultrasonic pulse through the water path alone was first recorded (the reference signal), then the sample was aligned parallel to the transducer surface to get the maximum amplitude of the signal (the bone signal).

All signals were filtered and amplified by a pulser/receiver unit (5900PR, OlympusCorp., MA, USA), viewed using an oscilloscope (54642A, AgilentInc., USA), and digitized at the 8bit resolution of the analog-to-digital converter and then stored for data processing.

### Transmission measurements

The regions of interest (ROIs), referred to as total number of scan points, are summarized in Table 2. 5 ROIs were determined to be an adequate number for each specimen. For the translation of the specimen, the margin of the transducer was kept within the specimen boundaries to ensure that the predominant lobe of the ultrasonic wave only covered the cancellous bone.

**Table 2:** Acoustic Properties for five probes.

Probe(MHz)	Attenuation, dB/cm			nBUA, dB/(cm MHz)			Speed of sound, m/s		
	Mean	SD	Min.-Max.	Mean	SD	Min.-Max.	Mean	SD	Min.-Max.
0.5	7.68	4.06	0.58–14.22	28.7	6.37	18.82–41.93	1514.41	16.81	1500.91–1556.16
1	18.86	7.49	6.98–30.32	17.31	10.39	5.91–43.99	1508.61	4.97	1502.03–1518.23
1.5	29.96	11.23	12.60–44.96	20.7	6.94	7.47–38.12	1502.36	2.19	1500.20–1507.02
2.25	41.4	12.07	19.08–68.18	16.52	5.35	5.55–29.78	1500.6	0.59	1500.04–1502.44
3.5	52.96	15.33	32.04–87.29	12.46	5.16	10.36–16.45	1500.15	0.15	1500.00–1500.61

A standard substitution technique was used to measure the attenuation and phase velocity and the attenuation was calculated using[20]:

$$\alpha (f)=20\log_{10}\frac{A_{sig}(f)}{A_{ref}(f)}/l$$ (1)

where, A_ref_(*f*) and A_sig_(*f*) represented the amplitude spectrums (magnitude of the Fourier transform) of the reference and bone signal, respectively, and *l* was the sample thickness.

The normalized broadband ultrasonic attenuation(nBUA) was calculated as the slope of the linear regression line of Eq (1)within the bandwidth of the transducer using:

$$nBUA=\frac{\Delta \alpha (f)}{\Delta f}$$ (2)

The speed of sound(SOS) is an important parameter of the ultrasonic acoustic properties of biological tissue. Due to the physiological variation of cancellous bone in different areas, the SOS was different in each direction and is representative of the specimen characteristics.

The phase velocity (*ν* (*f*)) was determined using[20]:

$$v(f)=\frac{1}{\frac{1}{v_{w}}-\frac{\phi (f)}{2\pi f\cdot l}}$$ (3)

where, v_w_ was the SOS in water(1500 m/s) and *ϕ* (*f*) was the phase difference between the reference signal and the bone signal and could be obtained from a Fourier transform:

$$\phi (f)=Arctg\frac{A_{sig}(f)}{A_{ref}(f)}$$ (4)

### Backscattering measurements

A 10×8×2cm polished steel plate was placed below the bone. The incident signals first passed through the water then the bone, and then reflected off the polished steel plate and returned to the transducer. For repeated assessments,3–6 mm cancellous bone segments were removed and the probes were changed each time.

### Statistical analysis

All data are presented as mean±standard deviation (SD) and were analyzed using SAS 9.1 software. Differences between each group were compared with a one-way ANOVA followed by a least significant difference(LSD) test with homogeneity of variance, or Dunnett T3 with heterogeneity of variance for multiple comparisons. A p*-*value<0.05 was regarded as statistically significant.

## Results

### Quantitative ultrasonic characterization in transmission measurements

**Fig 2:**
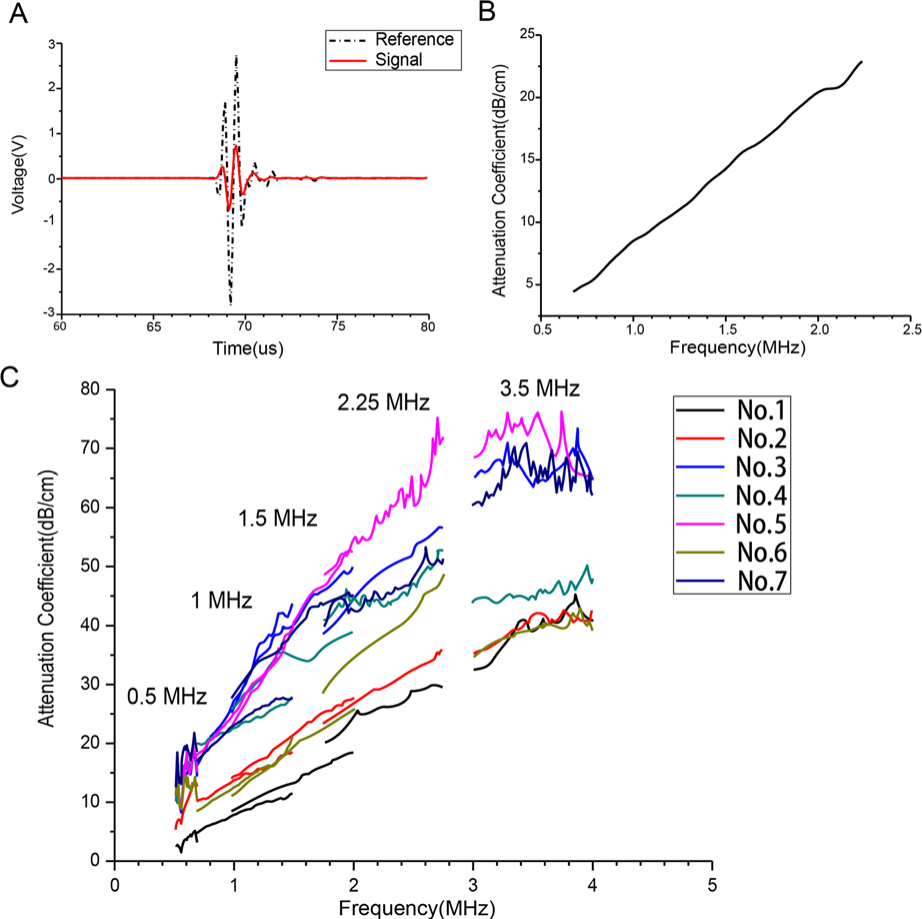
Relationship Between Attenuation and Frequency. (A) Reference signal(dashed line) and signal transmitted through bone(solid line) for the 1.5-MHz center-frequency transducer pair on specimen 1. (B) Relationship between attenuation and frequency for the 1.5-MHz center-frequency transducer pair on specimen 1. (C) Relationship between attenuation and frequency for 5 transducer pairs on 7 specimens.

Randomized typical wave forms that propagated through the water and specimen are represented by the dashed and solid lines, respectively, in Fig 2A.

The descriptive statistics of the acoustic properties for five probes are presented in Table 2. The mean, SD, and range are shown for the central-frequency attenuation, the nBUA, and the SOS. Specific data are shown in S1 Table, and results for the 7 ROIs measured by one transducer on 7specimensare pooled together and are represented as the last line.

To illustrate the relationship between the frequency and attenuation, a group of signals representing the spectrum and attenuation (the measurements ofthe1.5 MHz transducer onspecimen1 as an example)are manifested in Fig 2B. Theassociation between the attenuation and the frequency in the waveforms from the 5 transducer pairs from 7specimens was integrated and presented in Fig 2C. The correlation coefficients between attenuation and frequency were 0.38(0.5 MHz), 0.98(1 MHz), 0.86(1.5 MHz), 0.93(2.25 MHz), and 0.49(3.5 MHz).

**Fig 3:**
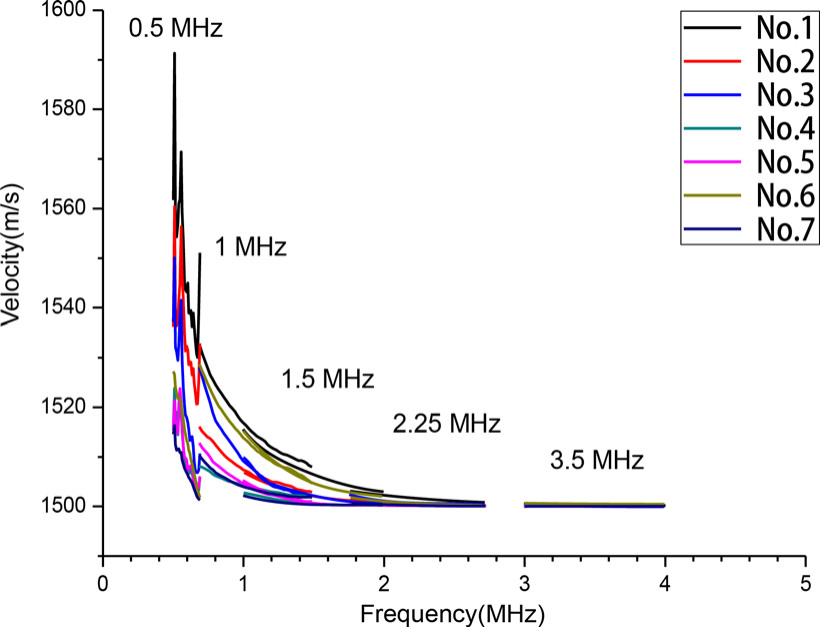
Relationship Between Velocity and Frequency. The speed of sound decreased as the ultrasonic frequency increased, which indicated a negative dispersion.

For the velocity measurements, linear regression was performed to manifest the type(positive or negative) and degree(slope) of the velocity dispersion. TheSOS decreased as the ultrasonic frequency increased in all ROIs, which indicates that the specimens featured negative dispersion. When the frequency of the transducers changed from 0.5 MHz to 3.5 MHz, the velocity dropped by about 14 m/s from 1514.41 ± 16.81m/s to 1500.15 ± 0.15m/s. Moreover, theSOSat 3.5 MHz(range:1500.00–1500.16m/s)was more stable than the SOS at 0.5 MHz (range:1501.67–1556.16m/s) (*p*<0.05)(Fig 3).

### A-Mode signal in transmission measurements

**Fig 4:**
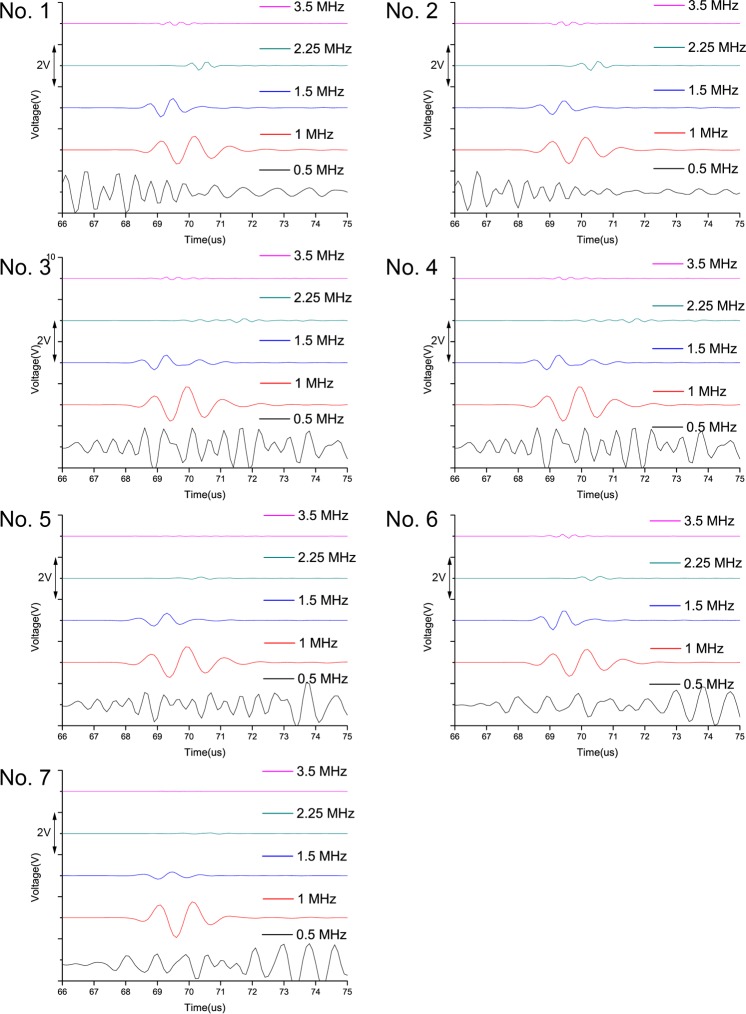
Ultrasound Attenuation of the Seven Specimens. For the same specimen, the ultrasound attenuation increased while the amplitude decreased with an increase in the frequency.

Generated by a transmitter, the A-Mode signal results were received and were illustrated in Fig 4. The normalized amplitudes were 0.24 ± 0.30(0.5 MHz), 0.24 ± 0.14(1 MHz), 0.12 ± 0.09(1.5 MHz), 0.05 ± 0.04(2.55 MHz), and 0.03 ± 0.03(3.5 MHz). There was a significant difference among the 1 MHz, 1.5MHz, and 2.25 MHz groups, such that the frequency increased and the amplitude attenuation decreased(*p*<0.05,*p* = 1,0.5 MHz *vs*. 1 MHz;*p* = 0.32, 0.5 MHz *vs*. 1.5 MHz;*p* = 0.23 2.25 MHz *vs*. 3.5 MHz).

### Depth of penetration in backscattering measurements

For the transducer at 0.5 MHz, obvious echo-signals were received at the initial thickness of the 7 specimens. The depths of penetration are shown in Table 3. Dunnett T3 was used for multiple comparisons due to heterogeneity of variance and no significant differences were found among the 5 groups (*p*>0.05).

**Table 3:** Depth of penetration at different frequencies.

Frequency(MHz)	Depth of penetration, cm							Mean(cm)	SD
	1	2	3	4	5	6	7		
0.5	2.40	1.80	1.90	2.00	2.00	2.60	2.60	2.19	0.34
1	1.60	1.50	0.90	0.90	1.20	1.50	1.20	1.26	0.29
1.5	1.60	1.50	0.70	0.60	0.90	1.50	0.60	1.06	0.46
2.25	0.90	1.00	0.70	1.50	0.60	1.90	0.60	1.03	0.50
3.5	1.20	1.00	1.40	1.10	0.60	0.60	0.90	0.97	0.30

### A-Mode signal in backscattering measurements

**Fig 5:**
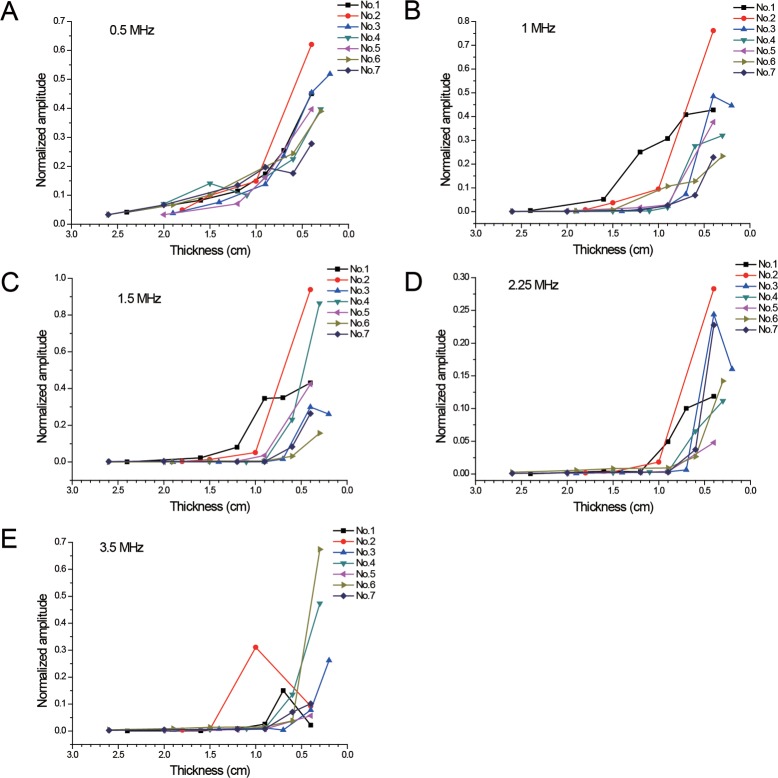
A-Mode Signal in Backscattering Measurements. Normalized amplitude at different specimen thicknesses for the 5 transducers was exhibited. The amplitude increased significantly when the thickness of the specimens reduced to about 0.6 cm.

The images in the time domain were obtained and the amplitudes of the signals were normalized. The polygrams of the normalized amplitude at different specimen thicknesses for the 5transducers are exhibited in Fig 5. The amplitude increased significantly when the thickness of the specimens was reduced to about 0.6 cm.

## Discussion

Our study showed that signal attenuation increased in response to an increase in the frequency. However, the nBUA at 1MHz, 1.5 MHz, and 2.25 MHz indicated no significant differences. The SOS descended rapidly within the frequency rangeof0.5–1.5 MHz, while its descending tendency alleviated when the frequency was higher than 2 MHz. For the backscattered measurements, the penetrating distances were recorded and the signals received at about 0.6 cm from the cortical bone increased significantly.

### Ultrasonic attenuation amplified with the increase of the frequency

Kantelhardt et al. [21, 22] used a 20 MHz intravascular ultrasound(IVUS) probe catheter placed in the pedicle to obtain intrapedicular images. Unfortunately, the near-total reflection of the ultrasound beam prevented the formation of images. As a result, they implied that using ultrasound transducers at frequencies greater than 20 MHz might not produce accurate images in bone. In our study (details in S1 Table), it was shown that attenuation at 3.5 MHz was 6.9 times that at 0.5 MHz, and 2.8 times that at 1 MHz. Mujagic [20] measured the attenuation at 3.5 MHz and found that it was amplified 2.3 times compared to the 1 MHz in human vertebral specimens.

Furthermore, Wear et al.[23] employed transducers at 200–300 kHz to 600 kHz to 1 MHz and found that the ultrasonic attenuation had a linear dependence on the frequency(correlation of 0.98–0.99). However, Strelitzki et al.[24]measured transmission on formaldehyde-fixed cadavericcalcaneus specimens, and found a non-linear relationship at frequencies below 400 kHz and a linear relationship above 800kHz.

In Fig 2C, the linear relationship between the attenuation and the frequency was obvious, especially on specimens 1, 2, and 6 that were diagnosed with acute osteoporosis. The correlation coefficients between attenuation and frequency were 0.38(0.5 MHz), 0.98(1 MHz), 0.86(1.5 MHz), 0.93(2.25 MHz), and 0.49(3.5 MHz). The attenuation coefficients displayed a fluctuation within the frequency bandwidth from 0.25 MHz to 0.5 MHz and a reserved tendency within 3.5 to 4.5 MHz. Langton *et al*.[25] reported that the linear dependence between attenuation and frequency occurred around 600kHz. Consequently, ultrasonic attenuation increased and the resolution improved with an increase in the frequency of the ultrasound probe and vice versa. For bone inspection, searching for transducers at the proper frequencies is important to balance attenuation with the image resolution.

### Speed of sound

The SOS can be used to predict the distance to the margin of the cortical bone. However, the velocity varied and fluctuated at different frequencies and therefore, it is important to find a specific ultrasonic frequency with a stable and accurate velocity value.

For ultrasonic diagnosis of bone tissue, the group velocity is usually measured. Since bone tissue is a complex, multiphase, porous, and anisotropic medium, two kinds of longitudinal waves(fast and slow) and one kind of transverse wave can be observed[26, 27]. Actually, the fast longitudinal waves are most commonly measured because the slow longitudinal waves have a greater dispersion and lower speed with a critical frequency of 1–10 kHz[28]. In trabecular bone, the methods of velocity measurement include time-domain and frequency-domain approaches. Time-domain methods often overestimate the speed[29],and therefore, a frequency-domain approach was applied in the current study in an attempt to provide more accurate results.

In our study, the velocity was about 1500 m/s and became stable for the transducers with frequencies above 2.25 MHz. The range of the SOS was 1501–1556 m/s for the transducer at 0.5 MHz, while the difference in the range was 0.61 m/s for the transducer at 3.5 MHz. It was shown that with an increase in the frequency, the SOS stabilized and its dispersion reduced(SD, 16.81(0.5 MHz), 4.94(1 MHz), 2.19(1.5 MHz), 0.59(2.25 MHz), and 0.15(3.5 MHz)). Fig 3 shows that the SOS descended rapidly within the frequency rangeof0.5–1.5 MHz, while its descending tendency alleviated as the frequency increased above 2 MHz.

In conclusion, at higher frequencies the probe may have a more stable SOS and resulted in better bone detection.

### Backscattering measurements

With respect to the transmission measurement results, the attenuation increased as the frequency increased, while the penetration distance decreased with an increase in frequency. In this study, the penetration distance was defined as the initial waves observed on the oscilliograph in a consistent condition such that the ultrasonic sonograph was kept in homeostasis, preventing weak signals from being recorded. Mujagic *et al*.[20]applied the Panametrics 5800PR (sonography) and the TDS 3012B (oscilloscope) in 7 vertebra and reported penetration distancesbetween1.65 and 1.90cm with no significant difference between the groups of the 1 MHz and 3.5 MHz transducers. Raphael et al.[15]observed a penetrating distance of 1.5cm with a 2.5MHz transducer in ovine vertebral bodies.

The backscattering measurements can anticipate the warning distance and Fig 5 showsthat when the bone sample got thinner at about 0.6 cm, it received abnormally increased signals and the amplitude of the waveform was directly viewed and easily recognized. Although the attenuation increased at higher frequencies, the abnormal waveforms were still identified. Raphael et al.[15]observed maximal backscatter amplitude at about 0.8cm,which was consistent with the findings of the current study.

### Limitations

Some limitations associated with the current study need to be clarified. First, isopropyl alcohol and methanol were used to remove the fat from the specimens; however, adipose tissue could significantly reduce the velocity and increase BUA. Therefore, the results presented here may have overestimated the SOS and underestimated attenuation[30, 31]. Secondly, the results showed considerable variation amongthe7 specimens, and the in vitro approach would add even more variation while this is a main problem for quantitative analysis. Though the specimens were obtained from fresh cadavers in this study, the results may not reflect the full microarchitectural variation of human vertebral cancellous bone. Thirdly, backscattered measurements may help in detecting cortical bone at a distance of 0.6cm. However, in some patients, the width of the pedicle is much smaller than 0.6cm[32] and this technique may have limited use in such patients. Furtherly, until now, no similar studies have been reported on animals or human beings in vivo. Thus, additional clinical studies should be carried out.

### Future investigation for the ultrasonic system

Possible circumstances where ultrasound could be applied should be considered. First, if a transducer can be integrated with the toolkit that drills the pilot hole, then the best trajectory could be searched for consecutively by the toolkit without changing the total surgical workflow[12]. Although patents have been granted for this idea[33, 34], they have been based on experimental studies as opposed to clinical practices. Second, simulations using the finite difference time domain method might help reduce the scale of experiments to determine the suitable frequencies[35, 36]. Finally, in combination with other techniques such as CAS navigational systems, the accuracy of ultrasonic navigation might be improved [37].

## Conclusions

With an increase in the frequency, the attenuation increased, the penetration distance decreased, and the stability of the SOS improved. For the backscattered measurements, at about 0.6 cm from the cortical bone, the warning signals could be observed easily. Ultrasound proved to be an effective, moveable, and real-time imaging system, however, it is unclear how ultrasound navigation will benefit pedicle screw insertion in spinal surgery, and future work is required. As the ultrasonic system is incorporated into the awl for the preparation of the screw hole, the ultrasound-guided pedicle screw implantation could be, theoretically, effective and promising.

## Supporting Information

S1 File Transmission Data file. (XLSX)

S2 File Backscattering Data file. (XLSX)

S1 Table (DOCX)
