# Supplementary material for: Basic Study for Ultrasound-Based Navigation for Pedicle Screw Insertion Using Transmission and Backscattered Methods
Source: PLoS One. 2015 Apr 10;10(4):e0122392. doi: 10.1371/journal.pone.0122392 (PMC4393101; doi:10.1371/journal.pone.0122392)
Supplement: S1 Table — (DOCX) [file pone.0122392.s003.docx]

Table. Acoustic Properties at 0.5 MHz

| No. | Attenuation, dB/cm | | | nBUA, dB/(cm MHz) | | | Speed of sound, m/s | | |
| --- | --- | --- | --- | --- | --- | --- | --- | --- | --- |
|  | Mean | SD | Min.-Max. | Mean | SD | Min.-Max. | Mean | SD | Min.-Max. |
| 1 | 1.06 | 0.44 | 0.58-1.76 | 22.01 | 3.36 | 18.82-27.19 | 1549.25 | 6.18 | 1539.67-1556.16 |
| 2 | 2.71 | 0.40 | 2.05-3.11 | 22.85 | 2.81 | 20.05-26.88 | 1523.74 | 3.82 | 1520.18-1530.10 |
| 3 | 8.13 | 1.01 | 7.30-9.71 | 36.13 | 4.38 | 30.54-41.93 | 1517.15 | 3.79 | 1511.43-1520.50 |
| 4 | 8.99 | 1.55 | 6.99-10.45 | 25.17 | 2.58 | 22.77-28.16 | 1503.48 | 2.02 | 1501.8--1506.30 |
| 5 | 11.03 | 1.65 | 8.23-12.35 | 31.25 | 6.03 | 23.32-39.46 | 1502.91 | 1.93 | 1501.67-1506.30 |
| 6 | 11.74 | 1.74 | 9.96-14.22 | 31.98 | 5.83 | 25.55-41.88 | 1502.27 | 1.09 | 1500.97-1503.61 |
| 7 | 10.12 | 0.69 | 9.04-10.95 | 31.50 | 3.77 | 25.26-34.47 | 1502.08 | 0.56 | 1501.50-1502.99 |
| Pooled | 7.68 | 4.06 | 0.58-14.22 | 28.70 | 6.37 | 18.82-41.93 | 1514.41 | 16.81 | 1500.91-1556.16 |

Table. Acoustic Properties at 1 MHz

| No. | Attenuation, dB/cm | | | nBUA, dB/(cm MHz) | | | Speed of sound, m/s | | |
| --- | --- | --- | --- | --- | --- | --- | --- | --- | --- |
|  | Mean | SD | Min.-Max. | Mean | SD | Min.-Max. | Mean | SD | Min.-Max. |
| 1 | 7.83 | 0.85 | 6.98-8.80 | 8.32 | 1.18 | 6.31-9.23 | 1516.86 | 1.34 | 1513.39-1518.23 |
| 2 | 13.71 | 1.39 | 11.54-15.04 | 10.51 | 1.83 | 8.81-12.50 | 1507.40 | 1.21 | 1503.31-1509.35 |
| 3 | 27.23 | 2.12 | 25.25-30.32 | 34.66 | 6.01 | 28.84-43.99 | 1509.18 | 1.29 | 1507.36-1510.40 |
| 4 | 22.26 | 4.99 | 17.23-28.76 | 10.37 | 6.39 | 5.91-14.29 | 1504.23 | 1.97 | 1502.03-1506.65 |
| 5 | 25.06 | 3.54 | 20.62-29.28 | 27.91 | 6.81 | 20.92-37.64 | 1504.65 | 1.48 | 1503.02-1503.69 |
| 6 | 12.50 | 1.12 | 13.59-15.41 | 15.41 | 6.05 | 9.37-22.70 | 1513.91 | 1.38 | 1512.59-1515.92 |
| 7 | 23.08 | 4.45 | 16.41-28.34 | 13.98 | 5.36 | 8.76-20.42 | 1503.98 | 1.97 | 1502.12-1507.24 |
| Pooled | 18.86 | 7.49 | 6.98-30.32 | 17.31 | 10.39 | 5.91-43.99 | 1508.61 | 4.97 | 1502.03-1518.23 |

Table. Acoustic Properties at 1.5 MHz

| No. | Attenuation, dB/cm | | | nBUA, dB/(cm MHz) | | | Speed of sound, m/s | | |
| --- | --- | --- | --- | --- | --- | --- | --- | --- | --- |
|  | Mean | SD | Min.-Max. | Mean | SD | Min.-Max. | Mean | SD | Min.-Max. |
| 1 | 13.47 | 0.77 | 12.60-14.14 | 11.25 | 4.00 | 7.47-17.68 | 1506.45 | 0.50 | 1506.60-1507.02 |
| 2 | 21.48 | 1.44 | 19.31-23.26 | 18.84 | 2.17 | 16.76-22.00 | 1502.02 | 0.36 | 1501.63-1502.57 |
| 3 | 40.43 | 3.79 | 35.53-44.19 | 27.48 | 3.25 | 22.37-31.31 | 1502.49 | 0.66 | 1501.87-1503.40 |
| 4 | 34.73 | 5.70 | 24.59-37.88 | 18.54 | 4.68 | 14.81-25.63 | 1500.86 | 0.68 | 1500.52-1502.07 |
| 5 | 40.57 | 4.36 | 34.48-44.96 | 27.35 | 7.11 | 20.30-38.12 | 1500.76 | 0.32 | 1500.47-1501.24 |
| 6 | 19.83 | 2.04 | 17.30-21.88 | 22.33 | 7.05 | 13.85-33.43 | 1504.82 | 0.91 | 1503.95-1595.98 |
| 7 | 39.12 | 5.34 | 33.22-47.37 | 19.09 | 3.89 | 14.71-23.57 | 1500.51 | 0.25 | 1500.20-1500.84 |
| Pooled | 29.96 | 11.23 | 12.60-44.96 | 20.70 | 6.94 | 7.47-38.12 | 1502.36 | 2.19 | 1500.20-1507.02 |

Table. Acoustic Properties at 2.25 MHz

| No. | Attenuation, dB/cm | | | nBUA, dB/(cm MHz) | | | Speed of sound, m/s | | |
| --- | --- | --- | --- | --- | --- | --- | --- | --- | --- |
|  | Mean | SD | Min.-Max. | Mean | SD | Min.-Max. | Mean | SD | Min.-Max. |
| 1 | 26.25 | 9.38 | 19.08-42.21 | 9.73 | 2.81 | 5.50-13.17 | 1501.51 | 0.89 | 1500.22-1502.44 |
| 2 | 29.30 | 3.99 | 25.32-33.88 | 13.81 | 2.72 | 9.24-16.55 | 1500.59 | 0.25 | 1500.32-1500.86 |
| 3 | 49.40 | 6.71 | 40.54-56.56 | 16.21 | 4.19 | 9.57-21.76 | 1500.95 | 0.45 | 1500.53-1501.61 |
| 4 | 43.06 | 2.35 | 40.37-46.13 | 16.76 | 3.71 | 12.56-19.68 | 1500.21 | 0.05 | 1500.15-1500.27 |
| 5 | 58.83 | 6.75 | 49.99-68.18 | 24.88 | 4.24 | 18.08-29.78 | 1500.10 | 0.06 | 1500.04-1500.74 |
| 6 | 38.38 | 1.29 | 36.90-40.71 | 18.12 | 3.52 | 15.67-24.30 | 1500.59 | 0.10 | 1500.47-1500.36 |
| 7 | 44.62 | 8.81 | 37.24-59.64 | 16.14 | 2.95 | 12.17-18.59 | 1500.22 | 0.12 | 1500.04-1500.35 |
| Pooled | 41.40 | 12.07 | 19.08-68.18 | 16.52 | 5.35 | 5.55-29.78 | 1500.60 | 0.59 | 1500.04-1502.44 |

Table. Acoustic Properties at 3.5 MHz

| No. | Attenuation, dB/cm | | | nBUA, dB/(cm MHz) | | | Speed of sound, m/s | | |
| --- | --- | --- | --- | --- | --- | --- | --- | --- | --- |
|  | Mean | SD | Min.-Max. | Mean | SD | Min.-Max. | Mean | SD | Min.-Max. |
| 1 | 39.12 | 3.76 | 34.18-44.51 | 14.03 | 4.13 | 8.56-18.71 | 1500.21 | 0.08 | 1500.11-1500.33 |
| 2 | 41.81 | 3.14 | 37.48-45.38 | 9.18 | 3.38 | 6.73-15.36 | 1500.09 | 0.03 | 1500.06-1500.14 |
| 3 | 63.55 | 8.77 | 57.70-78.91 | 15.75 | 7.77 | 9.93-26.54 | 1500.24 | 0.10 | 1500.07-1500.32 |
| 4 | 45.11 | 7.77 | 32.04-51.59 | 8.56 | 3.63 | 4.12-12.61 | 1500.14 | 0.15 | 1500.05-1500.41 |
| 5 | 74.15 | 4.12 | 67.03-77.62 | 15.10 | 3.74 | 10.99-20.75 | 1500.01 | 0.01 | 1500.01-1500.61 |
| 6 | 39.24 | 4.06 | 33.02-42.08 | 9.84 | 5.26 | 5.37-18.75 | 1500.37 | 0.15 | 1500.26-1500.61 |
| 7 | 67.72 | 11.08 | 60.68-87.29 | 14.88 | 2.62 | 5.39-18.75 | 1500.01 | 0.01 | 1500.00-1500.02 |
| Pooled | 52.96 | 15.33 | 32.04-87.29 | 12.46 | 5.16 | 10.36-16.45 | 1500.15 | 0.15 | 1500.00-1500.61 |
